# Effects of Bisphenol A on the Risk of Developing Obesity

**DOI:** 10.3390/nu16213740

**Published:** 2024-10-31

**Authors:** Mónica García García, Yolanda Picó, María Morales-Suárez-Varela

**Affiliations:** 1 Research Group in Social and Nutritional Epidemiology, Pharmacoepidemiology and Public Health, Department of Preventive Medicine and Public Health, Food Sciences, Toxicology and Forensic Medicine, Faculty of Pharmacy and Food Sciences, Universitat de València, Av. Vicent Andrés Estelles s/n, 46100 Burjassot, València, Spain; mogarga2@alumni.uv.es; 2 Food and Environmental Safety Research Group of the Universitat de València (SAMA-UV), Desertification Research Centre (CIDE) CSIC-GV-UV, CV-315 Km. 10, 7, 46113 Moncada, València, Spain; yolanda.pico@uv.es; 3 CIBER of Epidemiology and Public Health (CIBERESP), Carlos III Health Institute, Av. Monforte de Lemos 3-5 Pabellón 11 Planta 0, 28029 Madrid, Madrid, Spain

**Keywords:** bisphenol A, endocrine disruptor, epigenetics, obesity, obesogenic, metabolic syndrome

## Abstract

Background: Every year the global incidence of obesity increases considerably and among the factors that favor it is bisphenol A (BPA), an endocrine disruptor widely used in plastics and omnipresent in many everyday objects. Methods: A total of 19 studies published between 2018 and 2023 that addressed the relationship between BPA exposure and obesity were included in this review in order to better understand its behavior and mechanisms of action. Results: The studies reviewed conclude that BPA is an obesogen that alters the function of hormonal receptors, promotes metabolic syndrome, affects certain genes, etc., leading to a greater risk of developing obesity. With important emphasis on the ability to cause epigenetic changes, thus transmitting the effects to offspring when exposure has occurred during critical stages of development such as during gestation or the perinatal period. Conclusions: There is sufficient evidence to show that BPA is a risk factor in the development of obesity. Even so, further research is necessary to exhaustively understand the causal relationship between the two in order to develop prevention measures and avoid possible future adverse effects.

## 1. Introduction

Obesity is a chronic, recurrent, and multifactorial disease, as defined in the International Classification of Diseases [1]. Predisposing factors for obesity include lifestyle, genetic susceptibility, excess calories, etc. [2]. However, chemicals that act as endocrine disruptors (EDCs) also play a significant role. These chemicals are commonly found in the environment and in everyday products. Additionally, they are persistent substances, so humans are constantly and highly exposed to them. Therefore, special attention must be focused on this pathology and its causes to prevent and reduce new cases.

The Endocrine Society’s second Scientific Statement on endocrine disrupting chemicals defines EDCs as “an exogenous chemical or mixture of chemicals that interferes with any aspect of hormonal action” [3]. EDCs are compounds that can interfere with the correct functioning of the body, affecting hormonal regulation and metabolic homeostasis, which can lead to weight gain, among other effects [4]. Therefore, they suppose a risk for the development of obesity and related diseases, such as hypertension, dyslipidemia, type 2 diabetes mellitus, coronary heart disease, cerebrovascular disease, gallbladder disease, osteoarthritis, sleep apnea, and mental illnesses [5].

Bisphenol A (4,4′-(propane-2,2-diyl)diphenol), an endocrine disruptor, is a significant obesogen that promotes obesity. It is particularly notable as one of the first recognized EDCs; it was synthesized in 1891 and identified as an estrogenic agent in 1936 [6]. Its widespread use in the production of polycarbonates and epoxy resins [7,8] has favored its ubiquity in a multitude of everyday products. These include food and drink containers, linings of cans, toys, baby bottles, dental implants, and shopping receipts [9,10]. The major issue with BPA is its ability to migrate from packaging into food and beverages, especially when the packaging is subjected to high temperatures, reused, or manipulated [11].

In humans, the main route of BPA exposure is oral (ingestion), but it can also be absorbed through inhalation from the air and through the skin [12]. However, exposure routes to BPA vary significantly across different population groups due to differences in lifestyle, occupation, and vulnerability related to developmental stages. Infants and young children are among the most susceptible groups, with exposure often occurring through food and drink containers, toys, and even breastfeeding if BPA has accumulated in the mother’s body [13,14,15,16,17]. Adults are frequently exposed to BPA through dietary intake (plastics, cans, and receipts), occupational environments (manufacturing, dental offices), and consumer products [18,19]. Occupational exposure is more prominent among factory workers involved in plastic production, recycling, and cashiers frequently handling thermal paper receipts containing BPA [20,21,22,23]. Older adults can also be vulnerable due to their prolonged cumulative exposure, which may compound risks for metabolic diseases, heart conditions, and cognitive decline due to BPA’s effect on hormone regulation and cellular stress responses. Socioeconomic factors also influence exposure levels; communities with limited access to BPA-free products and higher reliance on processed or packaged foods may face elevated exposure.

Once BPA is absorbed into the body, it binds to hormonal receptors, including estrogen receptors (ER-alpha and ER-beta), androgen receptors (ARs), the thyroid receptor (TR), progesterone (PR), and retinoic acid receptors (RARs). It can also bind to the peroxisome proliferator-activated receptor (PPAR-y), retinoid X receptor (RXR), toll-like receptors (TLRs), and NOD-like receptors (NLRs). Additionally, it interacts with membrane receptors such as GPR30, or orphan receptors like the aryl hydrocarbon receptor (AhR) [11,12,24,25]. Other key mechanisms by which BPA influences obesity are through inflammation and the activity of its metabolites [26,27].

Previous studies have shown the different plausible pathways and mechanisms through which the binding of BPA to hormonal receptors significantly contributes to obesity development [28]. For example, in the case of BPA binding to ER-alpha and ER-beta, it initiates abnormal activation of pathways that regulate adipogenesis, the process of forming new fat cells [29]. This binding leads to dysregulation of adipose tissue development and contributes to obesity and metabolic disorders. 

In particular, BPA exposure influences the peroxisome proliferator-activated receptor gamma (PPARγ), a critical regulator of lipid metabolism and adipocyte differentiation [30]. Through this interaction, BPA can enhance lipid accumulation in adipocytes and stimulate differentiation of precursor cells into mature fat cells, contributing to an increased fat mass. Additionally, BPA’s interaction with estrogen receptors can interfere with leptin and adiponectin signaling [31,32], two hormones involved in energy balance and insulin sensitivity, further promoting metabolic dysfunction and increasing susceptibility to weight gain and obesity.

The effects of BPA differ notably between males and females, largely due to its endocrine-disrupting properties that interact differently with the hormonal systems in each sex. It has been shown to have a more pronounced impact on females compared to males primarily due to BPA’s structural similarity to estrogen that allows it to bind to estrogen receptors impacting estrogen-regulated processes more prominently. While the evidence suggests that BPA exposure adversely affects females more than males, it is important to consider that males also experience significant health risks from BPA, particularly concerning behavioral and developmental outcomes [33,34]. These sex-based differences in BPA’s effects are likely rooted in variations in hormonal receptor sensitivity, metabolic processing, and developmental pathways between males and females [35,36,37].

Previous studies indicate that BPA exerts effects even at low concentrations [28,38,39,40,41], as well as how small doses of BPA can produce stronger effects than larger exposures. This “low-dose paradox” (nonmonotonic dose response) is characteristic of EDCs [42]. In the case of BPA, it mimics hormones like estrogen, which naturally function at very low concentrations within the body. At low levels, BPA can interact more effectively with estrogen receptors and other hormonal pathways, leading to significant activation or inhibition of various metabolic and developmental processes, particularly those related to adipogenesis, neurodevelopment, and reproductive health [43,44]. In contrast, at higher doses, cellular receptor saturation or feedback mechanisms, such as downregulating receptor availability or activating metabolizing enzymes, can work to counterbalance excess hormonal-like stimulation and may mitigate the disruptive effects, reducing BPA’s biological activity.

In a systematic review carried out by Chunxue Yang et al. (2018) [45], it was demonstrated that early exposure to BPA is associated with an increased risk of developing obesity in adulthood, as shown in both epidemiological and laboratory studies. In a cohort study by Mustieles et al. (2019) [46], involving 298 children aged 9 to 11, BPA was observed to increase the risk of obesity, particularly abdominal obesity, in peripubertal children. A narrative review by Biemann et al. (2021) [5] revealed that BPA increased the risk of developing obesity, even at low concentrations. Therefore, there is sufficient evidence to support the importance of BPA in the development of metabolic diseases such as obesity.

Exposure to EDCs during critical developmental stages, such as the perinatal period and childhood, poses a significant risk of developing obesity in adulthood. This risk extends to both the exposed individual and their offspring [47]. For instance, BPA has been detected in the placenta, amniotic fluid [48], and human breast milk [49]. Therefore, exposure can occur intrauterine and after childbirth [5].

Due to the issues associated with BPA and its adverse effects, restrictions on its use in certain products have increased, and regulations become stricter. In 2012, the US Food and Drug Administration (FDA) prohibited the use of BPA in baby products [50]. In 2023, the EFSA (European Food Safety Authority) reassessed the health risks associated with BPA and decreased the tolerable daily intake (TDI) from the 4 µg/kg body weight per day established in 2015 to 0.2 ng/kg body weight per day [51]. Consequently, alternatives have been sought to replace BPA in plastic production, such as bisphenol S (BPS, 4,4′-sulfonyldiphenol) and bisphenol F (BPF, Bis(4-hydroxyphenyl methane)).

**Figure 1:**
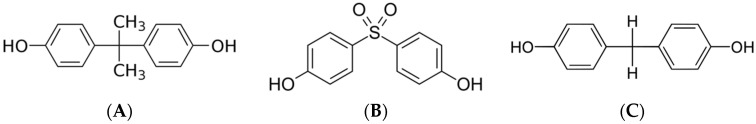
Chemical structure of bisphenol A (**A**), bisphenol S (**B**), and bisphenol F (**C**).

However, adverse effects similar to those of BPA have been observed due to their similar structure and characteristics (see Figure 1). In a narrative review of the most relevant epidemiological studies on the relationship between exposure to BPS and BPF and the incidence of obesity, Alharbi et al. found that both BPS and BPF contribute to the development of obesity through estrogenic and androgenic mechanisms similar those of BPA [10]. In a cross-sectional study carried out in the USA by Jacobson et al., urinary concentrations of BPS and BPF were associated with the development of abdominal obesity in children and adolescents. Additionally, BPF concentrations were associated with overweight and increased body mass index (BMI) [52]. Similar to BPA, the relationship between exposure to BPS and BPF with the risk of developing obesity is confirmed, therefore, it is important to consider BPA substitutes. This review included these substitutes to provide as much information about BPA as possible.

Although there is substantial scientific evidence demonstrating the influence of these EDCs on obesity, many aspects such as the mechanisms of action, exposure to mixtures, the behavior of these substances, the mode of exposure, etc., remain partly unknown. Therefore, this review aims to consolidate evidence from epidemiological, in vitro, in vivo, and in silico studies, selected based on specific quality criteria, regarding the impact of BPA on obesity development. Summarizing this evidence will help shed light on the gaps in knowledge that still exist regarding the mechanisms of action and adverse effects of these compounds.

## 2. Materials and Methods

### 2.1. Search Strategy

A literary search was carried out in three different databases: PubMed, Scopus, and EMBASE. Different studies were included, without differentiation by age or sex, focusing on an endocrine disruptor, bisphenol A (BPA), and its effects on obesity. The references of the identified articles were also reviewed for additional sources. To guide the search for articles, keywords were used that related obesity to bisphenol A as the main ECD. Specifically, “Obesity” and “Bisphenol A (BPA)” were used as keywords as follows:

((BPA) OR (Bisphenol)) AND (Obesity).

### 2.2. Inclusion Criteria

Studies that presented information on the relationship between exposure to bisphenol A and the risk of developing obesity were included.

Also included were articles that mentioned both diabetes and obesity, with a focus on obesity. Additionally, studies that included bisphenol A along with other endocrine disruptors were considered, focusing attention on BPA.

### 2.3. Exclusion Criteria

Articles were excluded for the following reasons:

- They focused on other contaminants or other disorders that were not related to metabolic disorders, especially obesity.
- They were in a language other than English.
- They did not include bisphenol A as an endocrine disruptor related to obesity.
- They were not articles published in the selected databases or in the selected period (articles were selected from 2018 to 2023).

The routes of exposure, duration, and life stage at which the individuals were exposed to BPA were considered, since they are determining parameters in its effects.

**Figure 2:**
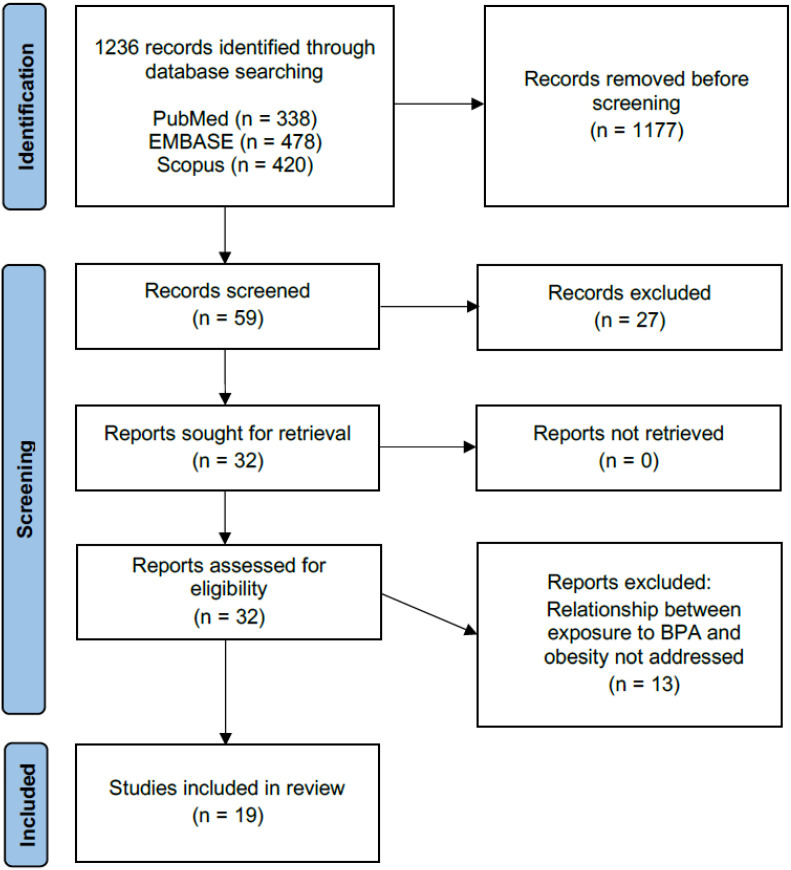
Study selection flow diagram.

Finally, 19 studies were selected. Figure 2 shows the flow diagram, following the PRISMA selection scheme [53,54].

### 2.4. Study Selection and Characteristics

As previously mentioned, the article selection process, following the inclusion and exclusion criteria outlined in the methodology, is shown in the flow diagram (Figure 2). Study selection was carried out by two of the authors independently and disagreements were resolved by discussion.

Initially, a total of 1236 articles were identified across three databases: PubMed, EMBASE, and Scopus. No additional databases were used. Duplicates were eliminated, and both titles and abstracts were evaluated according to the selection criteria. Of the articles identified, those that included BPA as an endocrine disruptor related to obesity were included in a first stay and based on the title and abstract, selecting a total of 59. After eliminating those articles that were duplicates, 32 remained, of which 13 did not meet the established inclusion criteria, since they focused on other contaminants and/or diseases that were not BPA and/or obesity; so, after making a more exhaustive review of the text, they were left with a final 19 articles included in this review.

They were grouped by type into narrative reviews [2,5,10,11,24,25,47,55], cross-sectional studies [52,56,57], cohort studies [46,58,59,60], and systematic reviews [45,61,62,63], described in Table 1, Table 2, Table 3 and Table 4, respectively.

The inclusion of articles from different categories makes the collected information much more complete, as it allows the problem to be observed from different perspectives. On the one hand, narrative and systematic reviews synthesize the information related to BPA and obesity, discussing possible mechanisms of action, all supported by multiple epidemiological, in vivo, and in vitro studies. On the other hand, cross-sectional and cohort studies provide evidence of cases of people affected by exposure to BPA and the development of obesity at a certain time, providing a temporal sequence between exposure and outcome.

### 2.5. Data Extraction

The data extracted from the different articles included the publication date, author names, title, study design, exposure assessment methods, and characterization of the results, as well as the statistical analysis and confidence in the results.

The studies were divided into four types of studies: narrative reviews, cross-sectional studies, cohort studies, and systematic reviews.

### 2.6. Quality Assessment of the Studies

The Newcastle–Ottawa Scale (NOS) was used to assess the quality of the cohort studies, evaluating the selection, comparability, and results of the studies [64].

The quality assessment criteria for cohort studies are divided into eight items, grouped into three categories. In the first category (selection), four items are evaluated: representativeness of the exposed cohort, selection of the non-exposed cohort, determination of exposure, and demonstration that the outcome of interest was not present at the beginning of the study. Each item in this category can be awarded a maximum of one star. The second category (comparability) includes a single item. If the comparability was considered based on the design or analysis, up to a maximum of two stars can be awarded. And finally, in the third category (outcome) three items were considered: the evaluation of the results, whether the duration of the follow-up was sufficient to obtain results, and the suitability of the follow-up of the cohort; in this category, each item can also be awarded a maximum of one star.

In this way, the maximum score a cohort study can achieve is 9 stars (4 stars for selection + 2 stars for comparability + 3 stars for outcome). Those studies with a score of 8–9 are considered very good, 6–7 is good, 1–5 is satisfactory, and 0–3 is unsatisfactory.

Similarly, for cross-sectional studies, an adaptation of the NOS was used, which has 7 items instead of 8 that are grouped in the same categories [24]. In the selection category, four items are considered: the representativeness of the sample, the sample size, the non-respondents, and the ascertainment of the exposure. For the comparability category, only one item is considered: the comparability of subjects in different groups based on design or analysis. Finally, in the outcome category, the two items that are considered are assessment of outcome and statistical test. The assignment of stars is different from cohorts, since in the selection category, a maximum of 5 stars can be obtained, in the comparability category, up to a maximum of 2, and in the outcome category up to a maximum of 3 stars.

The maximum score that each cross-sectional study can obtain is 10 stars (5 stars for selection + 2 stars for comparability + 3 stars for outcome). Studies with a score of 9–10 are considered very good, 7–8 is good, 5–6 is satisfactory, and 0–4 is unsatisfactory.

Table 5 includes the results of the quality assessment of the cohort and cross-sectional studies included in the NOS.

## 3. Results

From the selected articles, the evidence of the effect of BPA as an obesogen is summarized.

### 3.1. Narratives

In the present review, eight narrative reviews were included, and all of them agree on the role of BPA as an obesogen.

In the review carried out by Bansal et al. (2018) [24], four possible mechanisms of action of BPA related to obesity are described. These include its interaction with various hormonal receptors, such as modulating the estrogen receptor (ER), antagonizing the peroxisome proliferator-activated receptor (PPAR-y), stimulating or inhibiting toll-like receptors (TLRs), and activating the assembly of the NLPR3 inflammasome at NOD-like receptors (NLRs). Another mechanism is the alteration of the intestinal microbiome through the activation of the aryl hydrocarbon receptor (AhR), which is crucial for regulating fat storage, the intestinal capacity to extract energy from food, modulation of hormone levels that regulate appetite, and alteration of inflammatory pathways. BPA can also increase the risk of obesity through oxidative stress and elevated levels of proinflammatory cytokines. It activates the NFkB, increases cell death, reduces insulin secretion in primary murine pancreatic islets, and is associated with mitochondrial dysfunction. Finally, BPA is capable of altering the circadian rhythm, affecting metabolic homeostasis, which can lead to metabolic disorders such as obesity.

Moreover, Gupta et al. (2020) [2] also agree that BPA acts as an obesogen, either by activating the PI-3 kinase enzyme and causing an accumulation of triglycerides and lipoprotein lipase (LPL), as demonstrated in studies with rodents, or by acting as an agonist of the GPR30 membrane receptor and controlling the gene expression of estrogen receptors. BPA is also capable of acting on the thyroid axis, affecting biomarkers related to obesity such as adiponectin, leptin, and ghrelin. Furthermore, BPA increases the number and size of adipocytes by regulating the expression of the fatty acid binding protein 4 (FABP4), cluster of differentiation 36 (CD36), and protein convertase 1 (PCSK1) genes, altering adipocyte metabolism. Finally, BPA leads to obesity-related metabolic syndrome by reducing the release of adiponectin, which affects lipid metabolism and fatty acid oxidation.

Biemann et al. (2021) [5] demonstrate that there is epidemiological, in vivo, and in vitro evidence that BPA is related to obesity. BPA acts as a metabolic stress factor, which, adding to the fact that it is capable of causing epigenetic changes, results in a greater risk of developing obesity in offspring. This highlights the importance of avoiding exposure during critical stages of development, including gestation. BPA is capable of acting by binding to all types of receptors: estrogen receptors, peroxisome proliferator-activated receptor PPAR-y, CCAAT enhancer binding proteins (C/EBPα and C/EBPβ), regulatory element binding factor steroid 1 (SREBF1), thyroid receptors (TRs), retinoid X receptor (RXR), mammalian rapamycin (mTOR), signaling pathways, etc., through which it promotes adipogenesis. In addition, it is also capable of binding to the insulin receptor and causing an increase in pro-inflammatory signaling, which results in a decrease in insulin in adipocytes.

The study conducted by Boudalia et al. (2021) [25] includes evidence from epidemiological, animal, and in vitro studies that demonstrate the relationship between blood levels of BPA and the onset of obesity. Early stages of development are critical windows to BPA exposure, as they promote body weight gain in adulthood, supporting the ability of BPA to induce epigenetic changes discussed in the review by Biemann et al. (2021) [5].

Similarly, Murro et al. (2022) [47] agree on BPA’s interaction in adipogenesis due to its lipophilic character, altering normal lipid metabolism and adipogenesis. Early exposure to BPA has been linked to fat gain, hypertriglyceridemia, and elevated levels of fatty acids in the blood. High concentrations of BPA in the urine during the gestational period of mothers have been shown to increase abdominal obesity in children, mainly in girls. It has also been observed how BPA influences the regulation of the expression of genes related to adiponectin secretion, promoting weight gain and insulin resistance. BPA may have sex-dependent effects due to the similarity in its structure with estrogens (mainly female steroid hormones), so it may initially appear that exposure to BPA will not produce the same effects in women as in men. BPA not only presents estrogenic effects, but also antiandrogenic effects, so it is capable of affecting the conversion of testosterone into estrogen, which confirms the hypothesis that the effects may be sex dependent, since low concentrations of testosterone in the blood decreases the risk of accumulating fat in women but causes weight gain in men. Finally, BPA also affects adipose tissue macrophages by increasing their self-renewal through binding to the liver X receptor alpha. BPA promotes proinflammatory patterns of macrophages through interferon regulatory factor 5 (IRF5) and may influence levels of adipose tissue inflammation, predisposing to atherosclerosis and cardiometabolic diseases.

Kursunoglu and Yurekli (2022) [11] include in their review the mechanisms of BPA through hormonal receptors, which coincide with those previously mentioned in other studies, such as the following: binding to PPAR-y causing its deregulation; increasing adipogenesis by stimulating the differentiation of preadipocytes; regulating genes such as fatty acid binding protein 4 (FABP4) and cluster of differentiation 36 (CD36), and promoting lipid accumulation; among others. They demonstrate the effect of BPA on adipogenesis, such as causing obesity and other metabolic disorders. They also explain two additional mechanisms by which BPA can act as an obesogen. The first involves altering the neuroendocrine system in the central nervous system by stimulating the release of Agouti-related peptide (AgRP) and neuropeptide Y (NPY), two hormones that induce appetite. The second involves BPA reducing intestinal short-chain fatty acids (SCFAs) and increasing systemic levels of lipopolysaccharides, leading to chronic low-grade inflammation and disruption of lipid homeostasis.

In the study carried out by Alharbi et al. (2022) [10], the results show that BPA, BPS, and/or BPF exert obesogenic effects mainly by promoting estrogenic or androgenic activities and alterations in the genetic expression of different markers related to adipogenesis, such as PPAR-y and C/EBPα. Furthermore, they promote oxidative stress and inflammatory statuses by affecting the regulation of proinflammatory cytokines such as TNF-α and interleukins (IL). Finally, they are also capable of altering lipoprotein lipase (LPL) and fatty acid binding protein 4 (FABP4). Some of the studies have shown that BPS could be more potent than BPA; an in vitro study showed that murine 3T3-L1 preadipocytes, lipid accumulation, and upregulation of adipogenic gene expression were more observed in cells treated with BPS than those treated with BPA, and BPS has also been observed to potentially promote more potent hyperglycemic effects than BPA.

Finally, Khalil et al. (2023) [55] support the influence of BPA on obesity. At a molecular level, BPA can cause dyslipidemia and oxidative stress, release proinflammatory cytokines and adipokines such as TNF-α and leptin, and activate certain transcription factors such as PPAR-y or binding protein 1c. It also affects the sterol regulatory element (SREBP-1c) and enzymes such as hormone-sensitive lipase (HSL), resulting in increased adipocyte differentiation and fat storage. At the cellular level, it causes dysfunctions in adipose tissue, pancreatic ß cells, liver, and skeletal muscle. BPA is related to obesity and body mass index (BMI) by altering the regulators of adipogenesis, leading to increased mRNA of 11b-hydroxysteroid dehydrogenase type 1 (HSD1), thus accelerating the adipogenesis process. BPA activates PPAR-y, which contributes to the sensitization of adipocytes to insulin, altering blood glucose homeostasis and favoring the development of obesity.

### 3.2. Cross-Sectional

A total of three cross-sectional studies were included, with a combined sample size of 6358 participants, ranging from 745 [56] to 1831 [57]. Participants’ ages ranged from 6 years to 19 years or older. The information was collected from samples in two different countries: the USA and Korea.

In the article by Liu et al. (2019) [56], levels of BPA, BPF, and BPS in urine were evaluated to determine if there was an association with the development of obesity. They demonstrated a positive relationship between exposure to BPF and an increased risk of obesity in children and adolescents. Additionally, BPA and BPF were associated with abdominal obesity in children, with a possible sex difference. Similarly, Jacobson et al. (2019) [52] studied the association between BPA, BPS, and BPF measured in urine and the risk of obesity. The results showed that BPS concentrations increased the prevalence of general and abdominal obesity, while BPF concentrations were associated with an increased risk of developing abdominal obesity and a higher BMI. However, BPA was not significantly associated with general obesity, abdominal obesity, or any other outcome related to increased body mass. Finally, the study carried out by Lee et al. (2021) [57] measured BPA biomarkers in urine to determine the possible relationship with obesity. Higher concentrations of BPA were associated with an increased risk of developing obesity, suggesting it may be a potential risk factor.

The three studies share similar characteristics and yield comparable results, providing greater certainty about the positive relationship between BPA or its substitutes and the development of obesity.

### 3.3. Cohorts

On the other hand, four cohort studies were included, with a total sample of 1262, ranging from 59 [58] to 430 [59]. Participant’s ages varied from 2 to 12 years of age. The samples were collected from Spain, Denmark, China, and the USA.

Mustieles et al. (2019) [46] determined BPA concentrations in urine samples to study the possible relationship with overweight and obesity, observing changes in fat percentage, waist circumference, and weight. The results showed that an increase in urinary BPA concentration was correlated with a higher BMI, increased overweight, and a greater risk of developing general and abdominal obesity. Children with higher BPA concentrations had higher values in the waist–height ratio; however, no associations were observed concerning fat percentage. Additionally, Choi et al. (2020) [58] analyzed methylation profiles, specifically 594 CpG sites associated with obesity, to assess prenatal BPA exposure in children. They concluded that prenatal exposure to BPA could influence the differential methylation of IGF2R (cg19196862) at 2 years of age, leading to obesity in the future. The study carried out by Guo et al. (2020) [59], which analyzed associations between maternal BPA exposure and childhood obesity, showed that high maternal urinary BPA concentrations were positively related to an increase in the waist-to-height index in children, indicating a higher risk of central obesity. Moreover, significant sex differences were observed, with BPA exposure in mothers mainly affecting girls. Finally, Gajjar et al. (2022) [60] demonstrated that urinary concentrations of BPS were positively associated with a higher percentage of body fat, while with BPA, the relationship was inversely proportional. In this study, urinary concentrations of BPA and BPS were not associated with serum concentrations of adiponectin or leptin.

### 3.4. Systematic Reviews

In the case of systematic reviews, a total of four articles were included, all addressing the evidence of the effect of BPA on obesity.

Yang et al. (2018) [45] agree with the narrative review articles regarding the mechanisms of action of BPA on obesity. BPA causes mitochondrial dysfunction, leading to increased accumulation of diacylglycerol and reactive oxygen species (ROS), particularly in insulin-resistant tissues. Consistent with previously included articles, BPA induces oxidative stress, some ROS such as hydrogen peroxide (H_2_O_2_) can promote adipocyte differentiation and lipid peroxidation, which contribute to obesity and metabolic syndrome. Additionally, ROS increase key transcription factors for adipogenesis, such as PPAR-y and C/EBPα. Finally, BPA can induce DNA methylation, resulting in epigenetic changes.

Exposure to BPA during early stages of development has been linked to obesity later in life. In the review by Kim et al. (2019) [61], it was found that groups most exposed to BPA had a higher risk of developing obesity in adolescence compared to less exposed groups. This suggests that BPA has a dose-dependent effect, although various factors such as age or the stage at which exposure occurs influence it, it seems that a greater effect is expected at higher doses. This is confirmed in an in vitro study where stem cells derived from adipose tissue were treated with different increasing concentrations of BPS and BPF; it was demonstrated that there is a dose-dependent increase in lipid accumulation, since the accumulation was greater with the higher dose received [10,65]. BPA increases the number of adipocytes by regulating the expression of the FABP4, CD36, and PCSK1 genes; decreases the release of adiponectin and other adipokines; and disrupts adipocyte metabolism, thus increasing the risk of developing obesity. However, in the comparison of the obese group with the normal group, there are no significant differences in BPA levels. Although is contradictory, this may be attribute to the fact that obesity can vary depending on many factors, and even if BPA is one of the risk factors, there is no absolute association between the two, since you can suffer obesity and not be due to exposure to BPA. This endocrine disruptor mainly produces long-term effects, so it is much more critical that exposure occurs during early stages of development rather than in adulthood. Although, by producing epigenetic effects, maternal exposure in adulthood, if it is during the gestation stage, would also be critical.

At the same time, Ribeiro et al. (2020) [62] concluded in their review that there is a positive association between exposure to BPA and general obesity, abdominal obesity, and overweight in children and adults. Given the complexity of the action of EDCs, which act through various physiological pathways, they emphasize the potential dangers of exposure to certain EDCs like BPA, especially for children. As mentioned previously, early stages of development are critical.

Finally, Wu et al. (2020) [63] also found a positive and significant relationship between exposure to BPA and the risk of obesity. This finding is consistent with numerous studies included in the present review, which link higher concentrations of BPA in urine to obesity. Regarding the mechanisms, Wu et al. (2020) [63] aligns with Kim et al. (2019) [61] in highlighting the inhibition of adiponectin and adipokine release as a factor contributing to abdominal obesity.

### 3.5. Quality Assessment of the Studies

The scores of each included article are represented in Table 5. The cohort studies received scores between six and seven, while the selected cross-sectional studies received scores of eight. Therefore, based on these results, the articles included in this review can be considered of good quality.

## 4. Discussion

Exposure to BPA is now almost inevitable, as it is omnipresent in a multitude of everyday products and food products, leading to constant human exposure. As a result, BPA has been detected in the urine of over 90% of individuals in various populations, indicating a near universal exposure [66,67,68]. There is much evidence that demonstrates the effects of this endocrine disruptor on health, including its role in obesity.

Most studies agree on the interaction of BPA with various hormonal receptors, such as estrogen receptors, androgen receptors, progesterone receptors, thyroid receptors, corticosteroid receptors, PPAR-y, TLR receptors, NLR receptors, aryl hydrocarbon receptors (AhRs), GPR30 membrane receptors, retinoid X receptors (RXRs), retinoic acid receptors (RARs), liver X receptors (LXRs), and farnesoid X receptors (FXRs), all of which promote obesity.

BPA causes insulin resistance by interfering with insulin-activated receptors, increasing pro-inflammatory signaling and reducing insulin in adipocytes [5,47]. It inhibits the release of adiponectin while increasing pro-inflammatory cytokines in adipocytes and macrophages. It also increases the number of adipocytes by regulating the expression of genes such as fatty acid binding protein 4 (FABP4), group of differentiation 36 (CD36), and PCSK1, which promotes lipid accumulation [2,11,47]. Additionally, BPA inhibits the release of adiponectin and adipokine [61,63] and it causes a dysfunction in the metabolism of adipocytes, thus increasing the risk of developing obesity.

The potential interactions between the mechanisms of action of BPA that contribute to obesity should also be considered. As seen in the evidence reviewed, the main mechanisms of action could be grouped into three main categories: endocrine disruption, inflammatory pathways, and adipogenesis and fat accumulation. The nature of each of the mechanisms makes it almost impossible for it to not stray into one or both of the other categories, as they are closely interrelated. For example, BPA mimicking estrogen leads to metabolic dysregulation and adipogenesis, while pro-inflammatory cytokine production aggravates insulin resistance and disrupts fat metabolism. This serves to highlight the complexity of BPA’s action in the body and the difficulty in establishing a clear and concise causal pathway with obesity.

The effects of BPA on obesity are characterized by a nonmonotonic dose–response relationship, indicating that the impact of BPA does not follow a straightforward linear pattern. This implies that BPA may influence adiposity differently across varying dosages, with low and high exposures potentially affecting metabolic pathways uniquely. However, while the predominant evidence supports a nonmonotonic dose–response relationship for BPA and obesity, some studies indicate inconsistencies, particularly in specific populations, highlighting the complex, dose-dependent nature of BPA’s role in obesity development.

The studies reviewed suggest that the effects of BPA exposure on obesity are sex-dependent, with significant differences in adiposity and metabolic outcomes based on sex. This is evidenced by various studies highlighting distinct physiological responses in males and females, suggesting that sex plays a crucial role in the obesogenic effects of BPA. Sex-dependent factors like hormone levels, receptor expression, and fat distribution may influence how BPA impacts obesity differently in males and females. Estrogen receptor sensitivity to BPA, for example, could result in higher susceptibility to weight gain in females. While the evidence strongly supports sex-dependent effects of BPA on obesity, it is essential to consider that individual responses may vary based on genetic background and other external factors, indicating a complex interplay in the obesogenic potential of BPA.

Exogenous factors such as lifestyle, diet, or concurrent chemical exposures can significantly influence BPA’s obesogenic effects. For instance, diets high in fats and sugars appear to amplify BPA’s impact on obesity by accelerating lipid storage and insulin resistance, as BPA can disrupt metabolic tissues similar to natural estrogens, even at low doses [69]. A sedentary lifestyle lacking adequate physical activity may further enhance BPA’s obesogenic impact due to less efficient metabolic regulation. Collectively, these external factors can have a synergistic effect that heightens BPA’s obesogenic impact.

It must be noted that some of the studies reviewed found weak/no association between BPA and obesity, which could be due to several key factors. Methodological variability, such as differences in BPA measurement techniques, can impact result consistency. BPA is metabolized and eliminated relatively quickly in the body, so not all exposure assessment methods may be able to assess chronic exposure, potentially underestimating associations. Additionally, variations in age, sex, and metabolic health among participants introduce heterogeneity in the sample and can obscure associations in population studies. Moreover, a previous meta-analysis has shown that external factors might mask BPA’s effects by interacting with BPA exposure or by confounding its impact [61]. Lastly, early-life BPA exposure might show subtle effects that only manifest much later in life, complicating causality assessments.

Most studies agree that early stages of development, such as the perinatal period, are critical and highly susceptible periods to BPA exposure. BPA can induce changes in DNA methylation and produce epigenetic changes [45], with potential obesogenic effects that can be transmitted to offspring. Therefore, it is crucial to avoid BPA exposure during pregnancy.

Due to legislative restrictions on BPA, substitutes like BPS and BPF have appeared. However, many studies have shown that they are not a viable solution, as they exhibit similar effects on obesity and, in some cases, are even more potent than BPA [10,30]. Both BPS and BPF can influence hormonal pathways critical for reproductive health, with effects on estrogenic and androgenic receptors leading to altered hormone levels, reproductive organ abnormalities, and developmental disruptions in both males and females [70,71,72,73]. BPS has shown comparable potency to BPA in disrupting thyroid hormone regulation, which can have cascading effects on metabolism, growth, and neurodevelopment [74,75]. BPF is capable of binding to estrogen receptors and altering the production of adiponectin [56]; it also interferes with the hormonal regulation of RNA [74,75] and produces adverse effects in the hypothalamic–pituitary–gonadal axis [70,76,77,78], affecting the balance between energy consumption and saving. Furthermore, it may promote adipocyte differentiation and increase lipid accumulation through the activation of peroxisome proliferator-activated receptor alpha (PPAR-α) [79,80].

Importantly, BPS and BPF also demonstrate greater environmental stability [81,82], meaning they can persist longer in the environment, leading to prolonged exposure and potentially cumulative health risks. Due to these similarities and persistent effects, the increasing use of BPS and BPF in consumer products raises concerns that these alternatives may not be safer than BPA and may even exacerbate health and environmental risks.

### Methodological Considerations

As with any review, there are some methodological considerations for both the original studies and the review itself that must be considered to correctly interpret the results and findings. First, BPA effects strongly depend on doses, exposure routes, duration, and life stage; therefore, these should ideally always be taken into account when studying the effects of BPA exposure. This information is not always available in the reviewed studies, and this can affect the strength of the results. Another consideration is that the available evidence reviewed comes from a heterogeneous set of data from both preclinical and epidemiological studies with a wide variety of protocols and differences, such as in the exposure routes and the comparison with real BPA-free controls for example. In this respect, while the provided analysis points to BPA as (always) obesogenic, further studies in the field are needed to fully sustain this finding.

## 5. Conclusions

This review synthesizes 19 articles that relate BPA exposure to obesity. Most studies conclude that there is a positive association between the two, suggesting that BPA may be a risk factor for the development of obesity, which is added to lifestyle habits, caloric intake, and genetic susceptibility.

The general results of studies that analyze the relationship between exposure to BPA, BPS, and BPF together with obesity show a greater association in the case of BPA, but this does not mean that BPS and BPF do not carry the same, or even greater, risks as has been proven in many studies. This is because BPS and BPF have emerged as substitutes for BPA in an effort to reduce exposure to BPA and the effects derived from it; so, being relatively new, they have not been used for the same amount of time and thus, the exposure remains much smaller. For this reason and given that even after appearing much later they present the same or even greater effects, it is necessary to minimize exposure to these bisphenols, including BPA, to prevent possible adverse effects.

Given the evidence from studies on the role of BPA as an obesogen, it is important to carry out further and more exhaustive research on the mechanisms of action for BPF and BPS, since it has been shown in several studies that they generate the same effects on obesity given their great similarity to BPA. This includes investigating exposure to mixtures and not just isolated compounds, as well as the possible transgenerational epigenetic impacts these EDCs may have. This approach would help identify possible biomarkers at early stages and establish a true causal relationship between BPA exposure and the risk of developing obesity that could help in future prevention efforts.

## Figures and Tables

**Table 1:** Description of narrative reviews of exposure to BPA related to obesity.

Authors and Year	Aim of Study	Methodology	Results	Conclusions
Bansal et al. (2018) [24]	Understand the role of the immune system in mediating metabolic health and provide evidence of the effects of EDCs, such as BPA and phthalates, on the immune system and their impact on metabolic health	A narrative review of in vitro and in vivo studies supporting the influence of EDCs on the immune system and their association with the risk of metabolic diseases	BPA acts as an obesogen by binding to and altering the function of hormone receptors, causing dysbiosis in the intestinal microbiome, increasing oxidative stress, and disrupting the circadian rhythm, which can lead to metabolic disorders such as obesity	Constant and high exposure to EDCs such as BPA further increases the risk of developing metabolic diseases. Alteration of the immune system is considered a new mechanism linking EDCs exposure to metabolic dysfunction, due to their role as immunomodulators. For this reason, although more research is needed to support the hypothesis, the immune system should be considered a target for developing new treatment and prevention strategies against environmentally induced metabolic diseases
Gupta et al. (2020) [2]	Provide evidence of the role of obesogens in obesity in animals and humans, including their mechanisms of action. Additionally, propose sustainable strategies to prevent obesity	A narrative review of the effect of chemicals that act as obesogens in animals and humans, and their relationship with obesity	BPA acts as an obesogen by activating the PI-3 kinase enzyme, mimicking the action of the GPR30 receptor and estrogen receptors, and also affecting the thyroid axis. It has been shown to alter the metabolism of adipocytes, increasing their number and size by regulating the expression of certain genes. Additionally, BPA is associated to metabolic syndrome, related to obesity	Exposure to obesogens during critical stages of development has been shown to cause more severe obesity-related adverse effects in adulthood. It is essential to implement strategies to mitigate these effects, develop risk analyzes to identify high-risk groups, and better understand the factors related to detoxification and the metabolic pathways involved. One proposal is to use biomarkers. Additionally, more research is needed to reduce or replace them and thus avoid exposure
Biemann et al. (2021) [5]	Analyze the impact of BPA, along with other EDCs, on obesity and obesity-related metabolic disorders. Pay special attention to the effects of prenatal exposure to obesogens and strategies to identify, regulate and replace endocrine disruptors	A narrative review of the potential effects of BPA on obesity, with a special focus on prenatal exposure and strategies to reduce exposure to EDCs	Direct exposure to BPA positively regulates adipogenic, lipogenic, and inflammatory pathways, which may contribute to adipose tissue dysfunction and increase the risk of obesity. BPA acts through the estrogen receptor, PPAR-y, sterol regulatory element binding factor 1 (SREBF1), thyroid receptor (TR), retinoid mammalian rapamycin (mTOR) signaling, and the insulin receptor	There is sufficient evidence to show that EDCs such as BPA increase the risk of obesity. These can act at low concentrations and are often encountered as part of complex mixtures. They bioaccumulate in certain tissues, and exposure during the early stages of development is particularly critical. So, these chemicals must be avoided as much as possible
Boudalia et al. (2021) [25]	Identify the relationship between early exposure to EDCs and the development of obesity in adulthood	A narrative review of studies linking exposure to certain EDCs, including BPA, with the onset and development of obesity	BPA can bind to various hormone receptors and act as an obesogen. Epidemiological studies have observed a correlation between BPA levels in urine and blood and the appearance of obesity in adults. Animal studies show that uterine exposure to BPA is related to an increased body weight in adulthood, with a greater effect in females and a dose-dependent effect. Additionally, in vitro studies have shown that BPA affects the expression of adipocyte differentiation genes in 3T3-L1, without altering triglyceride accumulation, even at very low doses	Epidemiological, in vivo, in vitro, and in silico studies have linked exposure to EDCs, including BPA, with the development of obesity. Causing an increase in the number and size of cells, altering the regulation of adipose tissue, affecting hormones, regulating appetite, altering energy balance favoring the accumulation of calories, inducing insulin sensitivity, and affecting lipid metabolism
Murro et al. (2022) [47]	Know the possible mechanisms through which EDCs are related to obesity as possible risk factors	A narrative review on the mechanisms of action of EDCs involved in the development of obesity	BPA alters the differentiation and proliferation of adipocytes in murine cell lines and mesenchymal stem cells, as well as activates PPAR-y, increasing the expression of genes that promote the storage of fatty acids in adipocytes. Furthermore, BPA increases the self-renewal of macrophages by binding to the liver X alpha receptor in the liver, and these, in the presence of BPA, present a dose-dependent inflammatory response	Exposure to EDCs, such as BPA, can modify metabolism, increasing the predisposition to develop obesity by altering the hormonal system and affecting the development and regulation of adipose tissue, as well as the inflammatory system. Exposure to EDCs mixtures in humans has not been studied, which would be beneficial, as their combined models of action can cause synergistic or antagonistic effects, as demonstrated in studies, especially in pre-clinical observations, where the effects of BPA were contradictory to those of phthalates
Kursunoglu and Yurekli (2022) [11]	Show the clinical evidence and mechanisms by which EDCs act as obesogens and diabetogens	A narrative review of clinical and mechanistic evidence on EDCs as obesogenic and diabetogenic	BPA can (i) bind to PPAR-y and cause its deregulation; (ii) increase adipogenesis by stimulating the differentiation of preadipocytes into mature adipocytes; (iii) prevent adiponectin release and increase proinflammatory cytokines; (iv) regulate some genes such as FABP4 and CD36; (v) stimulate the release of Agouti-related peptide (AgRP) and neuropeptide Y (NPY); (vi) decrease the level of proopiomelanocortin (POMC); (vii) reduce short chain fatty acids and increase systemic lipopolysaccharide levels	Exposure to EDCs that act as obesogens and diabetogens is quite intense. Exposures during the perinatal stage are crucial, as they produce permanent and transgenerational effects. These exposures promote adipogenesis and fat accumulation, alter lipid metabolism, and affect the feeling of satiety. Although more studies are needed to validate the information
Alharbi et al. (2022) [10]	To summarize the epidemiological evidence of the association of urinary concentrations of BPS and BPF with the incidence of obesity and diabetes	A narrative review of the most relevant epidemiological studies on the relationship between exposure to BPS and/or BPF and the incidence of obesity and diabetes	The BPS and BPF had similar effects to BPA in terms of obesity, promoting estrogenic and/or androgenic activity, in addition to altering the genetic expression of different biomarkers related to adipogenesis. Producing imbalances in glucose and lipid homeostasis, leading to increased fat accumulation. Even some of the studies showed that BPS could be more powerful than BPA	Both BPS and BPF promote the development of obesity and diabetes. The mechanisms of action are primarily due to its estrogenic and androgenic activities, which alter the expression of adipocyte genes, induce oxidative stress, and trigger an inflammatory state. The effects are different depending on sex. More studies are needed to fully understand all possible mechanisms of action
Khalil et al. (2023) [55]	Highlight and evaluate the relationship between environmental pollutants and metabolic disorders such as obesity and diabetes, as well as explain the mechanisms and identify possible prevention methods	Database review between May and August 2022 in PubMed, ScienceDirect, ReadCube Papers, and Google Scholar and using Boolean logic to find articles related to environmental pollution and metabolic disorders. The references of the identified articles were also reviewed	BPA influences obesity through molecular mechanisms such as oxidative stress, transcription factors that regulate gene expression and play a role in the development of obesity, and through certain enzymes such as hormone-sensitive lipase (HSL), responsible for breaking down fat molecules stored in adipose tissue. As well as through cellular mechanisms such as dysfunctions of adipose tissue, pancreatic β cells, liver, and skeletal muscle	There are several mechanisms by which environmental pollutants, such as BPA, contribute to obesity and diabetes. However, the results related to direct pathogenesis are inconsistent because they are multifactorial diseases, making it difficult to know exactly the risk factor. Furthermore, there is a lack of understanding the impact of pollutants when present together or in mixtures. More research is needed to enhance our understanding of these contaminants and to develop strategies to reduce and prevent the increasing of obesity

Abbreviations: EDCs—endocrine-disrupting chemicals; BPA—bisphenol A; BPS—bisphenol S; BPF—bisphenol F; ER—estrogen receptor; Erα—estrogen receptor alpha; Erβ—estrogen receptor beta; PPAR-y—peroxisome proliferator-activated receptor; TLRs—toll-like receptors; NLRs—NOD-like receptors; AhR—aryl hydrocarbon receptor; TR—thyroid receptor; RXR—retinoid X receptor; NFKB—nuclear factor that enhances kappa light chains of activated B cells; LPL—lipoprotein lipase; GPR30—G protein-coupled receptor 30; FABP4—fatty acid binding protein 4; CD36—cluster of differentiation 36; PCSK1—proprotein convertase subtilisin/kexin type 1; IRF5—interferon regulatory factor 5; BATs—brown fat stores; AgRP—agouti-related peptide; NPY—neuropeptide Y; POMC—proopiomelanocortin; ILs—interleukins; HFD-induced—high-fat diet induced; C/EBPα—Ccaat-enhancer-binding protein alpha; C/EBPβ—Ccaat-enhancer-binding protein beta; TNF-α—tumor necrosis factor alpha; mTOR—mammalian rapamycin; SREBP-1c—sterol regulatory element; HSL—hormone-sensitive lipase; HSD1—hydroxysteroid dehydrogenase type 1; mRNA—messenger RNA; BMI—body mass index; WC—waist–hip ratio; NOAEL—no observed adverse effect level; NHANES—National Health and Nutrition Examination Survey.

**Table 2:** Description of cross-sectional studies of exposure to BPA related to obesity.

Author and Year	Location	Study Design	Participants	Exposure Measurement	Covariates	Results	Conclusions
Liu et al. (2019) [56]	USA (Iowa)	Cross-Sectional	745 children and adolescent aged 6–17 (NHANES 2013–2014)	Concentrations of BPA, BPF, and BPS in urine were measured by solid-phase extraction, high-performance liquid chromatography (HPLC), and tandem mass spectrometry	Demographic factors (age, sex, race/ethnicity), socioeconomic factors, lifestyle (sedentary lifestyle and diet), and adjustment of urinary creatinine levels	The odds ratio for overall obesity compared with the highest and lowest quartile of urinary BPA levels was 1.74 (95% CI, 0.92–3.31) for BPA, 1.54 (95% CI, 1.02–2.32) for BPF, and 1.36 (95% CI, 0.53–3.51) for BPS. The associations were greater in boys than girls for BPA and BPF. The results observed in abdominal obesity were similar	Exposure to BPA and BPF was positively associated with the risk of obesity in children and adolescents, being higher in boys than girls, suggesting a possible difference by sex
Jacobson et al. (2019) [52]	USA (New York)	Cross-Sectional	1831 children and adolescent aged 6–19 (NHANES 2013–2016)	BPA, BPS, and BPF in urine were measured with high-performance liquid chromatography (HPLC)-tandem mass spectrometry	Demographic factors (age, sex, race/ethnicity, educational level of the head of household, economics) and temperamental factors (time watching TV, caloric intake and exposure to tobacco smoke)	BPA was detected in 97.5% of the samples, BPF in 87.8% and BPS in 55.2%. Log-transformed urinary concentrations were associated with a higher prevalence of general obesity (OR, 1.16; 95% CI, 1.02–1.32) and abdominal obesity (OR, 1.13; 95% CI, 1.02–1.27). BPF detection was associated with a higher prevalence of abdominal obesity (OR, 1.29; 95% CI, 1.01–1.64) and continuous BMI z score (b = 0.10; 95% CI, 0.01–0.20)	Despite the difficulty of deducing a causal relationship between bisphenols and obesity, BPS and BPF concentrations were associated with abdominal obesity in children and adolescents, as well as BPF with overweight and an increase in BMI. BPA was not significantly associated with any body mass outcome
Lee et al. (2021) [57]	Republic of Korea (Seoul)	Cross-Sectional	3782 adult population aged 19 or older (KoNEHS 2015–2017)	BPA in urine was measured by liquid-liquid extraction and separation by high-performance liquid chromatography (HPLC) and mass spectrometry, followed by electrospray ionization and tandem mass spectrometry	Covariate standardized adjustment (CAS) of urinary dilution: urinary creatinine adjustment and specific gravity adjustment	BPA concentrations in the highest BPA quartile (OR, 1.62; 95% CI, 1.27–2.06) were positively associated with increased risk of obesity	The choice of the urinary dilution adjustment method can influence the results, the use of CAS can avoid problems in the association. Higher urinary BPA concentrations are linked to obesity and diabetes mellitus

Abbreviations: BPA—bisphenol A; BPS—bisphenol S; BPF—bisphenol F; NHANES—National Health and Nutrition Examination Survey; KoNEHS—Korean National Environmental Health Survey; HPLC—high-performance liquid chromatography; BMI—body mass index; WC—waist–hip ratio; CAS—covariate standardized adjustment; OR—odds ratio; CI—confidence interval.

**Table 3:** Description of cohort studies of exposure to BPA related to obesity.

Author and Year	Location	Study Design	Participants	Exposure Measurement	Covariates	Results	Conclusions
Mustieles et al. (2019) [46]	Spain (Granada)	Cohort	298 children 9–11 years	Total BPA in urine was determined by liquid chromatography and mass spectrometry	Age, sex, and urinary creatinine	An increase in log units of urinary BPA concentrations was associated with higher BMI (β = 0.22; 95% CI, 0.03–0.41) and with higher odds of being obese (OR, 1.46; 95% CI, 1.05–2.05). BPA was also associated with an increased risk of abdominal obesity (OR, 1.45; 95% CI, 1.03–2.06). The highest waist-to-height ratio values were found in children with higher BPA concentrations (β = 0.007; 95% CI, −0.001–0.015). No associations were found with body fat percentage	It was shown that BPA can act as an obesogen in peripubertal children, increasing the risk of obesity, especially abdominal obesity
Choi et al. (2020) [58]	Denmark (Copenhagen)	Cohort	59 children 2, 4, 6, and 8 years	Urinary BPA was measured during the second trimester of pregnancy (mean 20 weeks of gestation) and determined by HPLC chromatography and tandem mass spectrometry	Mother’s age at pregnancy, mother’s and father’s BMI when child was 2 years old, mother’s smoking status, mother’s educational level, premature birth, low birth weight, duration of breastfeeding and caloric intake of the child at 4, 6, and 8 years (not available at 2 years), all adjusted to the corresponding ages. Also adjusted for urinary creatinine	In the 2-year-old group with higher BPA content, cg19196862 (IGF2R) methylation levels increased significantly (*p* = 0.00030, corrected false discovery rate *p* < 0.10), however at 6 years, they did not. Increasing the standard deviation of IGF2R methylation at 2 years during ages 2 to 8 years significantly increased BMI by 0.49 (95% CI, 0.08–0.90) in girls but not in boys. The indirect effect of prenatal BPA exposure on BMI in early childhood through methylation at cg19196862 (IGF2R) at age 2 years was significant	Prenatal exposure to BPA influences differential methylation of ICG2R at age 2 years (sensitive period of DNA methylation), affecting BMI until later childhood. Furthermore, the effects are sex-specific
Guo et al. (2020) [59]	China (Jiangsu)	Cohort	430 children 3 and 7 years old And 190 mother–child pairs	Urinary BPA concentrations were determined by large-volume injection gas chromatography with tandem mass spectrometry	Urine creatinine, mother’s BMI before pregnancy, maternal education, annual family income, child’s sex, child’s age, duration of breastfeeding, and childhood passive smoking. Also, gestational weight gain, gestational age, and birth weight	Urinary BPA concentration in mothers was positively related to waist-hip ratio only in 7-year-old girls (b ¼ 0.508 cm; 95% CI, 0.067–0.950). The risk of central obesity related to BPA exposure was significantly higher in the second (OR, 1/4 2.510; 95% CI, 1/4 1.146–5.499) and third tertiles (OR, 1/4 2.584; 95% CI, 1/4 1.186–5.631) than in the first, (*p* for trend 1/4 0.022)	Prenatal exposure to BPA causes an increase in waist circumference in children, increasing the risk of central obesity at school age, predominantly in girls
Gajjar et al. (2022) [60]	USA (Ohio)	Cohort	233 children 8 years and 242 12 years (HOME Study 2003–2006)	Urinary BPA and BPS were determined by solid phase extraction coupled to HPL chromatography and isotope dilution tandem mass spectrometry with peak focusing	Child sex, physical activity, maternal BMI, sociodemographic factors (race/ethnicity, age, education, marital status, employment, and maternal and child insurance)	Each 10-fold increase in urinary BPA concentrations was inversely associated with percent body fat at 8 years (β = −1.2; 95% CI, −3.4–1.0) and 12 years (β = −1.6; 95 %CI, −4.0–0.9). However, urinary BPS concentrations were positively associated with percent body fat at age 8 (β = 1.1; 95% CI, −0.6–2.7) but not at age 12 (β = 0.1; 95% CI, −1.7–1.8). Urinary BPA and BPS concentrations were not associated with serum adiponectin or leptin concentrations	Urinary concentrations of BPA and BPS during childhood could not be related to an increased risk of childhood adiposity at 8 and 12 years of age

Abbreviations: BPA—bisphenol A; BPS—bisphenol S; HPLC—high-performance liquid chromatography; BMI—body mass index; OR—odds ratio; CI—confidence interval; ICG2R—insulin-like growth factor 2 receptor.

**Table 4:** Description of systematic studies of exposure to BPA related to obesity.

Author and Year	Aim of Study	Methodology	Results	Conclusions
Yang et al. (2018) [45]	Summarize the epidemiological and laboratory evidence related to early exposure to EDCs and obesity to understand the possible mechanisms that promote childhood obesity	Systematic review of epidemiological and laboratory evidence related to early exposure to EDCs, its mechanisms of action and childhood obesity	There is much evidence from epidemiological and laboratory studies indicating how early exposure to endocrine disruptors such as BPA contributes to the development of obesity in adulthood. This occurs through various mechanisms such as mitochondrial dysfunction, oxidative stress and DNA methylation	Early exposure, such as perinatal exposure, to endocrine disruptors such as BPA leads to a greater risk of developing obesity in adulthood, through different mechanisms such as mitochondrial dysfunction, oxidative stress and DNA methylation, as has been proven in multiple epidemiological and laboratory studies
Kim et al. (2019) [61]	Clarify the causal relationship between BPA exposure and childhood obesity	A systematic review was carried out along with two meta-analyses to understand the association between BPA exposure and the risk of obesity in children	The group most exposed to BPA had a significantly higher risk of childhood obesity than the group less exposed (OR, 1.566; 95% CI, 1.097–2.234, *p* = 0.014). Although the obese group did not show differences in BPA concentrations compared to the normal group (standardized mean difference = 0.166; 95% CI, −0.121–0.453, *p* = 0.257)	Epidemiological data demonstrate that BPA increases the risk of obesity in children. Although more studies are needed that relate the causal association between BPA and obesity at each stage of development and depending on sex
Ribeiro et al. (2020) [62]	Know the association between human exposure to certain EDCs, including BPA, and obesity	Systematic review and meta-analysis of exposure to endocrine disruptors and anthropometric measures of obesity	BPA was positively associated with general and abdominal obesity in children and adults, some studies suggesting that it was specific to age and sex. The meta-analysis indicated a significant association between BPA exposure and overweight (OR, 1.254; 95% CI, 1.005–1.564), obesity (OR, 1.503; 95% CI: 1.273–1.774), and with increased waist circumference (OR, 1.503; 95% CI: 1.267–1.783) in adults	There is a positive association between exposure to EDCs such as BPA and obesity. Although causality cannot be determined
Wu et al. (2020) [63]	Review epidemiological studies that related BPA exposure to obesity	Systematic review in different databases such as PubMed, Web of Science, and Embase to find articles that related BPA exposure to obesity	The combined OR of obesity risk for the highest level vs. the lowest of BPA exposure was 1.49 (95% CI, 1.38–1.61, I2 1/4 44.2%, P 1/4 0.016), so there is a positive correlation between BPA level and obesity. And a dose-response analysis showed how an increase in BPA by 1 ng/mL increased the risk of obesity by 11%, the results were similar for different types of obesity, gender, and age	Exposure to BPA may increase the risk of general and abdominal obesity, since it can inhibit the release of adiponectin and adipokine, a key protective factor against abdominal obesity. Although it cannot be determined that a causal relationship exists, more longitudinal studies are required

Abbreviations: EDCs (Endocrine-Disrupting Chemicals); BPA (bisphenol A); OR (Odds Ratio); CI (Confidence interval); PPAR-y (Peroxisome proliferator-activated receptor); C/EBPα (Ccaat-enhancer-binding protein alpha).

**Table 5:** Assessment of the quality of the cohort and cross-sectional studies included by the Newcastle–Ottawa scale.

Author and Year	Study Design	Selection	Comparability	Outcome	Total Score
Mustieles et al. (2019) [46]	Cohort	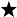 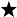 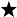	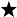	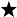 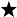	6/9
Choi et al. (2020) [58]	Cohort	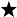 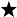 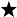	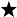	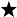 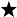 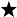	7/9
Guo et al. (2020) [59]	Cohort	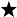 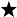 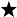	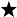	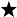 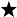 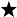	7/9
Gajjar et al. (2022) [60]	Cohort	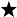 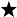 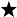	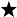	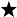 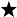 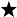	7/9
Liu et al. (2019) [56]	Cross-sectional	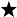 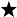 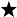	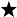 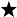	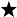 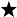 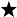	8/10
Jacobson et al. (2019) [52]	Cross-sectional	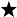 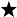 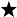	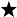 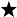	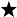 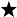 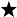	8/10
Lee et al. (2021) [57]	Cross-sectional	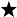 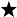 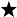	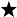 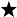	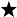 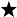 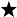	8/10
